# Supplementary material for: Clustering earthquake signals and background noises in continuous seismic data with unsupervised deep learning
Source: Nat Commun. 2020 Aug 7;11:3972. doi: 10.1038/s41467-020-17841-x (PMC7414231; doi:10.1038/s41467-020-17841-x)
Supplement: Supplementary file 2 — Supplementary Information [file 41467_2020_17841_MOESM2_ESM.pdf]

Clustering earthquake signals and background noises in continuous seismic data with unsupervised deep learning
Supplementary information

Léonard Seydoux *[1], Randall Balestriero[2], Piero Poli[1], Maarten de Hoop[3], Michel Campillo[1], and Richard Baraniuk[2]

[1]ISTerre, équipe Ondes et Structures, Université Grenoble-Alpes, UMR CNRS 5375, 1381 Rue de la Piscine, 38610 Gières, France
[2]Rice University, Electrical and Computational Engineering, 6100 Main MS-134, Houston, TX, 77005, USA
[3]Rice University, Computational and Applied Mathematics, 6100 Main MS-134, Houston, TX, 77005, USA

Received 30 July 2019; Accepted 13 July 2020; Published 07 August 2020 in *Nature Communications*
https://doi.org/10.1038/s41467-020-17841-x

Contents

Supplementary Note 1 2

Supplementary Note 2 2

Supplementary Note 3 2

Supplementary Figure 1: Learning results with different parameters 3

Supplementary Figure 2: Comparison of clusters D with P-wave microseismic energy 6

Supplementary Figure 3: Comparison of clusters C with Rayleigh wave microseismic energy 6

Supplementary References 6

*Corresponding author: leonard.seydoux@univ-grenoble-alpes.fr

Supplementary Note 1

In order to observe the within-cluster waveforms, we select the time segment of data starting at t_i and ending at $t_i + dt$, where dt is the temporal resolution of the scattering coefficients. The time segments are extracted with an additional small time delay ϵdt in order to allow for cross-correlating the time segments. We align the M waveforms $w_m(t)$ belonging to the same cluster with respect to a reference waveform $w_r(t)$ by means of cross-correlation, and collect the maximal correlation coefficient

$$c_{mr} = \max_{\tau} \int_{t=0}^{T} w_m(t) w_r(t-\tau) dt \tag{1}$$

Supplementary Note 2

We here discuss the different tests on the parameters used in the scattering network. One key parameter is the number of knots used to learn the shape of the wavelet. This parameter is responsible for the wavelet duration in time, and inherently for the wavelet bank quality factor. Indeed, a small number of knots defines a wavelet localized in time with a large frequency bandwidth and vice-versa. We therefore vary the number of knots in Fig. 5A to C in order to observe both the clustering and reconstruction losses onto a small subset of the dataset (8.5 hours). These tests are also very helpful to show that the procedure still works with a small amount of data (9 hours), a situation where deep convolutional neural networks are known to fail easily. We see that taking a low number of 7 knots (case A) allows to better reconstruct the input data with a loss of 4.20 (Table 1), but have a relatively high clustering loss (3.79). We observe in Fig. 5A that the cumulative curves trends are not clearly separated between clusters 2 and 3, also indicating that the clustering may have not converged to a stable description of the data. As we can see on Table 1 for cases A to C, increasing the number of knots (from 7 to 15) improves the clustering quality, but lowers the reconstruction loss. Even if the detection results are highly similar between cases A to C, we consider 11 knots to be a good trade-off between a high clustering quality and a reasonable reconstruction loss. In any case the precursory signals are always recovered even with a small amount of data, a clear advantage of our clustering procedure over clustering strategies based on classical deep convolutional neural networks.

We then conduct 3 additional tests onto daylong data, where the number of knots is fixed to 11, and where we investigate the pooling factor of the scattering layer which defines a trade-off between the stability of the scattering coefficients and the final time resolution of the analysis. A very large pooling value (case F) could lead to a degraded time resolution, but will still be able to detect seismic events that are very localized in time, and therefore the number of clusters is similar in cases D and F because the pooling factor is large enough. In contrast, a smaller pooling could lead to a smaller time resolution, without being stable enough for clustering (case E). With this choice of pooling factor, we observe that a larger number of clusters are kept after training with, which is a sign of instability. The clustering loss is high (3.67) in comparison with other clustering results. The pooling factor therefore must be chosen with respect to the maximal duration of interest, and should be maximized if no *a priori* on the signal in search is available.

The case D presented in detail in the present study (Figs. 3 and 4) has an intermediate pooling factor leading to a $\sim$32-sec final time resolution with three layers. In addition, we tested in case D a larger number of octaves and wavelets per octaves at each layer. This test presents the lowest clustering and reconstruction losses, which is mostly due to the presence of more filters at each layer to describe the data. Note that increasing the number of wavelet per octave do not change the number of parameters to be optimized in the learning procedure since the filter bank of each layer is derived from the learnable mother wavelet only.

Supplementary Note 3

We here explain how we compared the microseismic energy with the clustering results.

We collect the spectral pressure calculated from the WAVEWATCH III model [8] on a 0.5×0.5 degree grid globally, from 2017-06-01 to 2017-06-18. This pressure data cannot be directly used as a proxy for radiated seismic energy, because the radiation of body and surface waves depends on the bathymetric profile of the seafloor [9]. According to [9], the equivalent radiated spectral energy can be derived from the pressure with taking into account the resonance of the water column at each point of the grid as amplification factor. We therefore used the amplification model presented in [9], where the global bathymetry is taken into account. We then considered the source time function of each points of a 4×4 degree grid, and correlated it with the temporal within-cluster detection. Because the pressure data is availble every 3 hours, we decimated the within-cluster detection on the same time basis.

The correlation is tested for several frequency bands (0.1 to 0.2, 0.2 to 0.3, 0.3 to 0.45 and 0.45 to 0.6) and seismic waves (P waves, S waves and Rayleigh waves). For each frequency band, the maximally correlated source time function and seismic wave type is identified and represented in Fig. S2 and S3. In addition to the

water-column resonnance amplifications, we also apply different corrections for the different seismic wave types. The P-wave spectral energy is corrected from the shadowing of the Earth's core (no energy should be recorded between 104 and 140 degrees of epicentral distance). This first correction is applied as a mask on the correlation coefficients between within-cluster detection rates and source time functions. For Rayleigh waves, we also took into account the strong attenuation effects of the crust heterogeneities at these frequencies. We here considered an exponentially decaying attenuation with distance, with a decay of 1/500 km^{-1}.

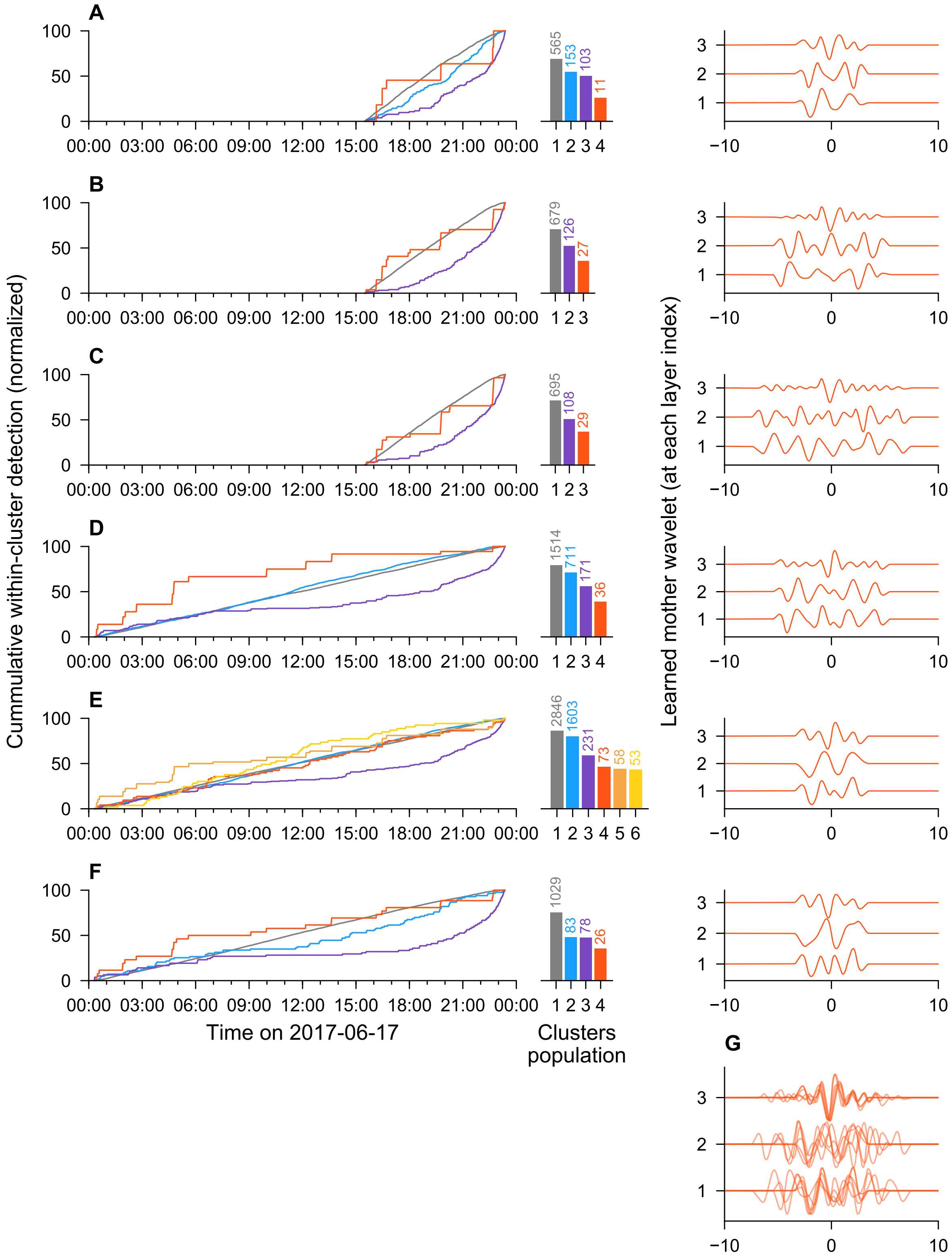

Supplementary Figure 1: Learning results with different parameters. The different parameter sets are given in Table 1. The left and middle plots respectively show the within-cluster cumulative detections and the within-cluster number of samples after 10,000 training epochs. The right plots show the final learned wavelets at each layer. **A–F**: results obtained with the parameters sets given in Table 1. The case D is the case analyzed in details in Fig. 3 and 4. **G**: learned mother wavelet at each layer with all parameter sets.

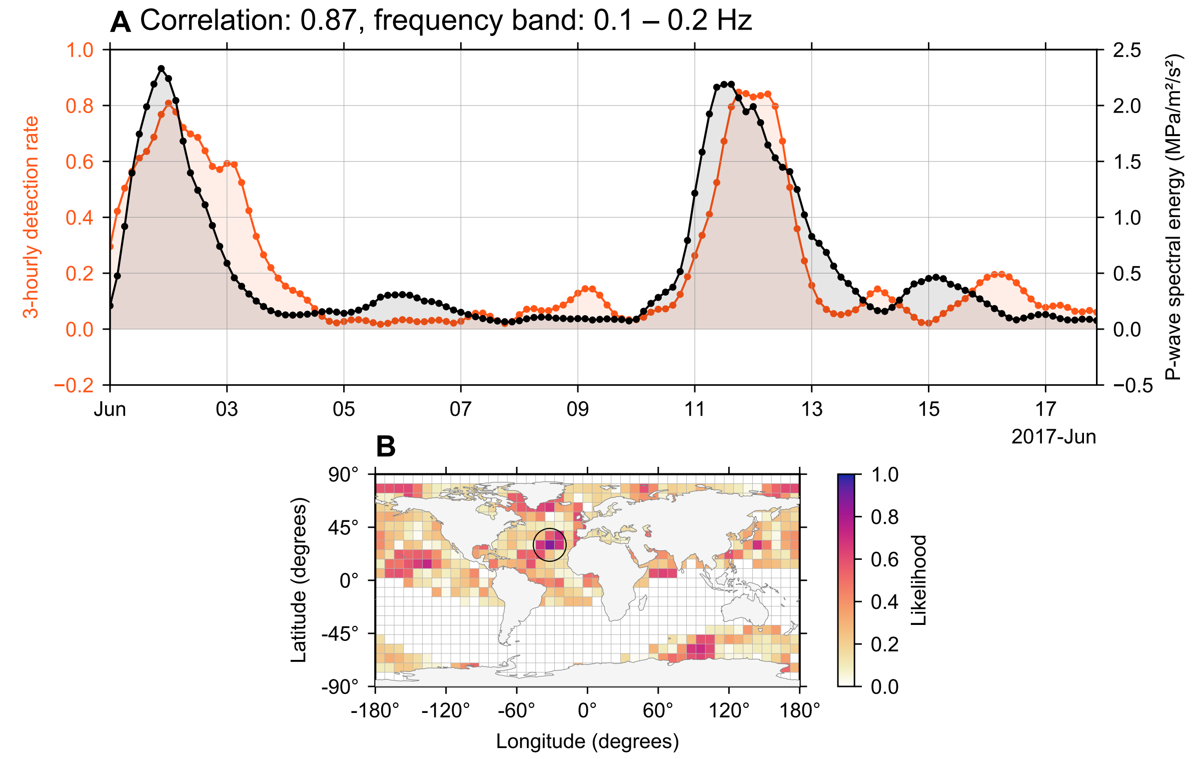

Supplementary Figure 2: Comparison of clusters D with P-wave microseismic energy. A: the whithin-cluster 3-hourly detection is presented in red curve over 17 days of 3-components seismic data. The best-matching radiated P-wave spectral energy in the frequency band 0.1 to 0.2 Hz is presented in black line. **B**: global matching likelihood of the spectral P-wave radiated energy between 0.1 and 0.2 Hz on a 4×4 degrees grid. The likelihood is corrected for theoretical P-wave shadow zones due to the presence of the core (between 104 and 140 degrees of epicentral distance), visible by the zero-likelihood zone. The highest likelihood from which the source-time function is extracted and presented in A is highlighted with a black circle in B.

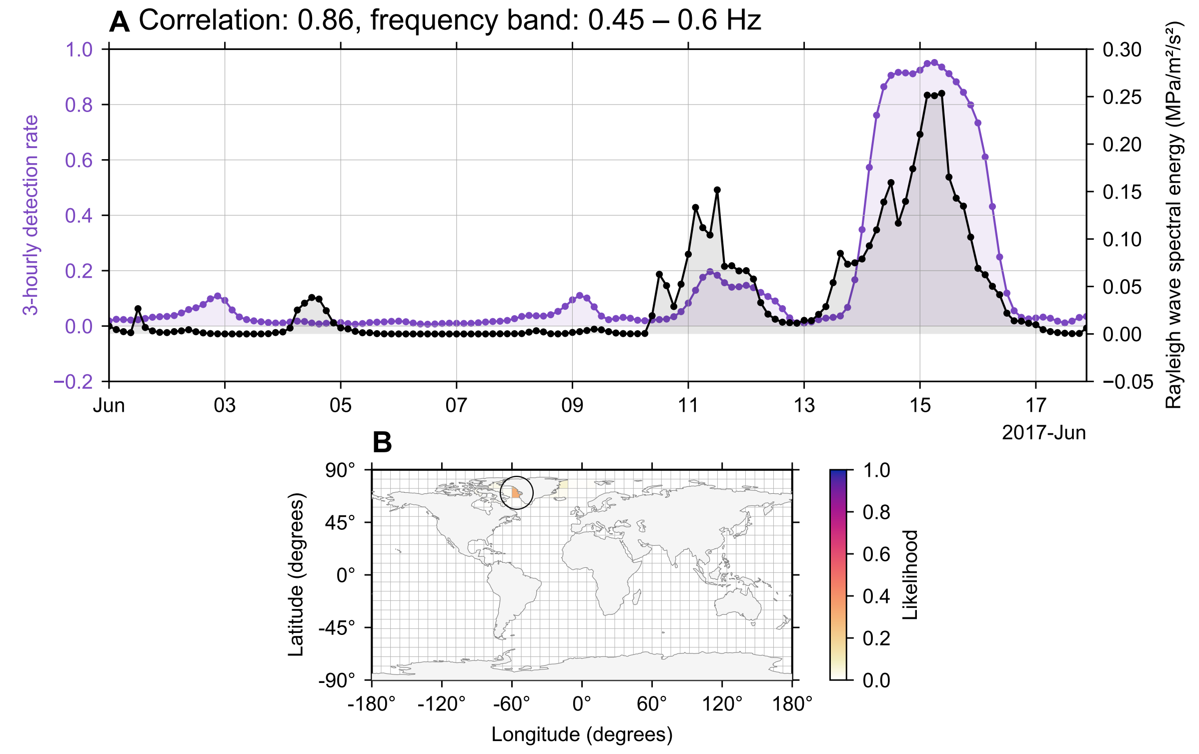

Supplementary Figure 3: Comparison of clusters C with Rayleigh wave microseismic energy. A: the whithin-cluster 3-hourly detection is presented in purple curve over 17 days of 3-components seismic data. The best-matching radiated Rayleigh-wave spectral energy in the frequency band 0.45 to 0.6 Hz is presented in black line. **B**: global matching likelihood of the spectral Rayleigh-wave radiated energy between 0.45 to 0.6 Hz on a 4×4 degrees grid. The likelihood is corrected from theoretical Rayleigh wave attenuation due to strong scattering at these frequencies. The highest likelihood from which the source-time function is extracted and presented in A is highlighted with a black circle in B.

Supplementary References

[1] Andén, J. & Mallat, S. Deep scattering spectrum. *IEEE Transactions on Signal Processing* **62**, 4114–4128 (2014).

[2] Andén, J. & Mallat, S. Scattering representation of modulated sounds. *15th DAFx* **9** (2012).

[3] Balestriero, R., Cosentino, R., Glotin, H. & Baraniuk, R. Spline filters for end-to-end deep learning. In Dy, J. & Krause, A. (eds.) *Proceedings of the 35th International Conference on Machine Learning*, vol. 80 of *Proceedings of Machine Learning Research*, 364–373 (PMLR, Stockholmsmässan, Stockholm Sweden, 2018).

[4] Dumoulin, V. & Visin, F. A guide to convolution arithmetic for deep learning. *arXiv preprint arXiv:1603.07285* (2016).

[5] Kingma, D. P. & Ba, J. Adam: A method for stochastic optimization. *arXiv preprint arXiv:1412.6980* (2014).

[6] Sifre, L., Kapoko, M., Oyallon, E. & Lostanlen, V. Scatnet: a matlab toolbox for scattering networks (2013).

[7] Xu, L. & Jordan, M. I. On convergence properties of the em algorithm for gaussian mixtures. *Neural computation* **8**, 129–151 (1996).

[8] Ardhuin, F. *et al.* Ocean wave sources of seismic noise. *Journal of Geophysical Research: Oceans* **116(C9)** (2011).

[9] Li, L., Boue, P. & Campillo, M. Spatiotemporal connectivity of noise-derived seismic body waves with ocean waves and microseism excitations *Eartharxiv* (2019).
